# Amelioration of Beta Interferon Inhibition by NS4B Contributes to Attenuating Tembusu Virus Virulence in Ducks

**DOI:** 10.3389/fimmu.2021.671471

**Published:** 2021-05-17

**Authors:** Wei Zhang, Miao Zeng, Bowen Jiang, Tong Lu, Jiaqi Guo, Tao Hu, Mingshu Wang, Renyong Jia, Dekang Zhu, Mafeng Liu, Xinxin Zhao, Qiao Yang, Ying Wu, Shaqiu Zhang, Xumin Ou, Yunya Liu, Ling Zhang, Yanling Yu, Leichang Pan, Anchun Cheng, Shun Chen

**Affiliations:** ^1^ Research Center of Avian Disease, College of Veterinary Medicine, Sichuan Agricultural University, Chengdu City, China; ^2^ Institute of Preventive Veterinary Medicine, College of Veterinary Medicine, Sichuan Agricultural University, Chengdu City, China; ^3^ Key Laboratory of Animal Disease and Human Health of Sichuan Province, Sichuan Agricultural University, Chengdu City, China

**Keywords:** duck TMUV NS4B, STING, beta interferon, immune evasion, virus replication

## Abstract

Our previous studies reported that duck Tembusu virus nonstructural protein 2A (NS2A) is a major inhibitor of the IFNβ signaling pathway through competitively binding to STING with TBK1, leading to a reduction in TBK1 phosphorylation. Duck TMUV NS2B3 could cleave and bind STING to subvert the IFNβ signaling pathway. Here, we found that overexpression of duck TMUV NS4B could compete with TBK1 in binding to STING, reducing TBK1 phosphorylation and inhibiting the IFNβ signaling pathway by using the Dual-Glo^®^ Luciferase Assay System and the NanoBiT protein-protein interaction (PPI) assay. We further identified the E2, M3, G4, W5, K10 and D34 residues in NS4B that were important for its interaction with STING and its inhibition of IFNβ induction, which were subsequently introduced into a duck TMUV replicon and an infectious cDNA clone. We found that the NS4B M3A mutant enhanced RNA replication and exhibited significantly higher titer levels than WT at 48-72 hpi but significantly decreased mortality (80%) in duck embryos compared to WT (100%); the NS4B G4A and R36A mutants slightly reduced RNA replication but exhibited the same titer levels as WT. However, the NS4B R36A mutant did not attenuate the virulence in duck embryos, whereas the G4A mutant significantly decreased the mortality (70%) of duck embryos. In addition, the NS4B W5A mutant did not affect viral replication, whereas the D34A mutant slightly reduced RNA replication, and both mutants exhibited significantly lower titer levels than the WT and significantly decreased mortality (90% and 70%, respectively) in duck embryos. Hence, our findings provide new insight into the development of attenuated flaviviruses by targeting the disabling viral strategies used to evade the innate defense mechanisms.

## Introduction

The innate immune response of the host is considered the first line of defense against viral infection. Once the virus invades, the pattern recognition receptors (PRRs) of the host cell, such as the acid-inducible gene I (RIG-I)-like receptor (RLR) family, recognize the viral nucleic acids, leading to the activation of the transcription factors NF-kB and IFN regulatory factors 3 and 7 (IRF3/7). The activation and nuclear translocation of these transcription factors subsequently induce the production of type I IFNs, which initiate the expression of ISGs that prevent viral infection (1, 2).

Duck Tembusu virus belongs to the genus *Flavivirus* family *Flaviviridae* and is an emerging pathogen associated with severe egg drop syndrome that has caused huge economic losses to the duck industry in China since 2010 (3–5). As with other flaviviruses, duck TMUV is a single-stranded, positive-sense RNA with a 10,990 bp genome. The open reading frame (ORF) encodes a unique polyprotein precursor that is subsequently cleaved by cellular and viral proteases into three structural proteins (core, membrane, and envelope) and seven nonstructural (NS) proteins (NS1, NS2A, NS2B, NS3, NS4A, 2KNS4B, and NS5) (6–8).

To replicate well in hosts, flaviviruses have to develop sophisticated strategies to evade or subvert the host innate immune response. Accumulated studies revealed that different NS proteins from the same flavivirus could antagonize IFN-β production through the similar cellular components in RIG-I signaling pathway. For example, ZIKV NS2A, NS2B, and NS4B inhibited IFN-β production through blocking TBK1 phosphorylation (9). DENV1/2/4 NS2A and NS4B commonly antagonized TBK1 phosphorylation and IFN-β induction (10). Furthermore, NS2A, NS2B, NS3, NS4A and NS4B proteins from KUNV could inhibit the phosphorylation and nuclear transduction of STAT2 (11). Similar to other flaviviruses, recent studies have reported that duck TMUV could also encode different NS proteins to inhibit IFN-β production by targeting the similar cellular components. Duck TMUV NS1 suppressed virus-triggered IFNβ expression by targeting VISA to disrupt the RLR pathway in HEK293 cells (12). Additionally, in our previous study, we analyzed the ability of the 10 proteins encoded by duck TMUV to block the IFN system and found that the expression of NS2A, NS2B, and 2KNS4B resulted in robust IFN signaling inhibition. We further found that duck TMUV NS2A inhibited IFNβ induction by targeting duck STING (13), and duck TMUV NS2B3 could cleave and bind duck STING to subvert the induction of IFNβ (14), suggesting that different duck TMUV proteins could commonly target the same cellular components through different strategies to suppress IFNβ production. However, the mechanism by which duck TMUV NS4B subverts the host innate immune response is unclear and requires further study.

Flavivirus NS4B is the largest hydrophobic NS protein and it shares the same predicted topology with five integral transmembrane segments (15). Increasing numbers of studies have provided evidence that it appears to play an important role in counteracting innate immune responses. Munoz-Jordan et al. first reported that Dengue virus (DENV) NS4B is involved in blocking IFN signaling by interfering with STAT1 phosphorylation (16). Subsequent deletion analyses suggested that the first 125 amino acids of DENV-2 NS4B are sufficient for the inhibition of IFN signaling (17). Likewise, the E22 and K24 residues in NS4B of West Nile virus (WNV) were shown to control IFN resistance in cells expressing subgenomic replicons (18). Moreover, HCV NS4B can interact with STING (stimulator of interferon gene, also known as MITA, MPYS, ERIS, and TMEM173) and disrupt the interaction of STING with downstream signaling effectors to block host antiviral immune responses (19, 20). A recent study also showed that the HCV-encoded NS4B protein inhibited TLR3-mediated interferon signaling by downregulating TRIF protein levels (21). These studies revealed that flavivirus NS4B developed a certain mechanism to evade host immune responses; however, the effect of this mechanism on viral replication has not yet been revealed.

In this study, we explored the role of NS4B as an IFN antagonist in the inhibition of host immune responses and the effects of its inhibitory function for the replication and virulence of duck TMUV *in vitro* and *in vivo*. Consistent with duck TMUV NS2A, we found that overexpression of duck TMUV 2KNS4B could also inhibit RIG-I-mediated IFN expression signaling by competitively binding to stimulator of interferon genes (STING) with TBK1, reducing TBK1 phosphorylation and suppressing IFN production and the effective phases of the IFN response. In addition, deletion analysis using a reverse genetics approach *in vitro* and *in vivo* showed that the first 38 amino acids of NS4B are responsible for the STING-NS4B interaction and its inhibitory effect on IFN signaling, which plays an essential role in viral replication and virulence. Our findings offer important insights into how duck TMUV establishes a mechanism to subvert the host innate immune response and they provide an approach for the development of attenuated viral vaccine candidates.

## Materials And Methods

### Ethics Statement

The animal studies were approved by the Institutional Animal Care and Use Committee of Sichuan Agricultural University (No. SYXK(川)2019-189) and followed the National Institutes of Health guidelines for the performance of animal experiments.

### Cells, Viruses and Antibodies

Duck embryo fibroblasts (DEFs) and BHK-21 cells were grown supplemented with 10% FBS and maintained in Dulbecco’s modified Eagle’s medium (DMEM) (Gibco Life Technologies, Shanghai, China). All cells were cultured at 37°C, 5% CO_2_. The duck Tembusu virus CQW1 strain (GenBank Accession: KM233707) was isolated by our laboratory (22), and the measured virus titer was 6.3×10^6^ TCID_50_/100 μL, which was reported previously (23). Antibodies (Abs) against Flag, His, Myc, and β-actin were purchased from TransGen Biotech, and rabbit anti-pTBK1 was purchased from Cell Signaling Technology. Mouse anti-duck TMUV antibodies were prepared by our laboratory.

### Plasmid Constructs

The sequence of the 2KNS4B gene was amplified from the duck TMUV CQW1 strain genome and cloned into the pCAGGS expression vector with a His tag at the C terminus using standard molecular biology techniques. The plasmids pCAGGS-duRIG-I, duMDA5, duMAVS, duTBK1, duIRF7, and duSTING-Flag and pBiT-LgBiT-duRIG-I, duMDA5, duMAVS, duTBK1, duIRF7, duSTING-Myc, pBiT-SmBiT-duTBK1 and STING-Flag were constructed as described in our previous study (13) for the NanoLuc Binary Technology (NanoBiT) assay. 2KNS4B and its truncations or mutants were cloned into pBiT2.1C-SmBiT with a Flag tag. Moreover, duSTING and its truncations or mutants were inserted into pBiT1.1N-LgBiT with a Myc tag. 2KNS4B fragments with different mutations were amplified by PCR using the duck TMUV replicon (pACYC-duck-TMUV-Replicon-NanoLuc, pAC-TVRep-Nluc) as a template and inserted into the pACYC- vector to construct NS4B mutations of the duck TMUV replicon and inserted into the pACNR- vector to construct NS4B mutations of the recombinant duck TMUV infectious clone.

### Real-Time RT-PCR

Total RNA was isolated from selected tissues using RNAiso Plus reagent. The quantity of RNA in each sample was determined using a NanoDrop 2000 (Thermo, Waltham, MA, USA), and RT-PCR was performed on each sample using a 5X All-In-One RT Master Mix Reagent Kit in accordance with the manufacturer’s instructions (Applied Biological Materials, Richmond, BC, Canada). Finally, the cDNAs were stored at –80°C until use. qPCR was used to detect the expression of duIFNβ in the samples. qPCR was performed using the Bio-Rad CFX-96 Real Time Detection System (Bio-Rad, USA). Threshold cycle (Ct) values were normalized to the housekeeping gene duβ-actin, and the relative expression levels of each target gene were calculated with the comparative Ct (2^-ΔΔCt^) method. The real-time PCR conditions and protocols have been previously described (24).

### Indirect Immunofluorescence Assay (IFA)

Transfected BHK-21 cells were washed three times with cold phosphate-buffered saline (PBS) and fixed with 4% paraformaldehyde overnight at 4°C. After three washes with PBS containing 0.1% Tween 20 (PBST) for 5 min each time, the cells were permeabilized with 0.2% Triton-X-200 for 30 min at 4°C and blocked with 5% bovine serum albumin (BSA) in PBS for 1 h at room temperature. Subsequently, the cells were washed three times with PBST and incubated with the primary antibody (diluted 1:2,000) for 2 h at room temperature in 1% BSA. Following three washes with PBST, the cells were incubated with a secondary antibody (diluted 1:5,000) for 1 h at room temperature in 1% BSA and then incubated with 4’,6-diamidino-2-phenylindole (DAPI) for 10 min. Finally, the coverslips were washed extensively and fixed onto slides. Fluorescence images were taken with a fluorescence microscope (Bio-Rad, USA).

### Luciferase Reporter Assay

Originally, the DEFs were seeded onto a 48-well plate and transiently cotransfected with the pGL3-IFNβ-Luc/pGL4-IRSE-Luc and pRL-TK plasmids. Subsequently, the cells were transfected with pACGGS-2KNS4B-His. Twenty-four hours later, the cells were challenged with 100 μL duck TMUV (containing 1000 TCID_50_). At 24 hpi, the cells were harvested for luciferase assays. For stimulation, pACGGS-2KNS4B-His was cotransfected with each component plasmid (pCAGGS-MDA5, RIG-I, MAVS, STING, TBK1 and IRF3-Flag) and reporter plasmid for 24 h. The luciferase activities were determined with a Dual-GloLuciferase Assay System (Promega) and normalized based on the Renilla luciferase activity.

### NanoLuc Binary Technology (NanoBiT) Protein-Protein Interaction (PPI) Assay

The NanoBiT PPI assay was performed as previously described (13). Briefly, two proteins with potential interactions were fused with the LgBiT or SmBiT subunit and transfected into DEF cells for 20 h, as described previously. PRKACA-SmBiT (protein kinase cAMP-activated catalytic subunit alpha) and PRKAR2A-LgBiT (protein kinase cAMP-dependent type II regulatory subunit alpha) were considered positive controls, whereas the LgBiT fusion plasmid coexpressed with HaloTag-SmBiT was used as a negative control. Subsequently, the luminescence value was measured according to the manufacturer’s instructions. Importantly, if the signal from the unknown PPI pair is less than tenfold higher than the negative control, this result may indicate a nonspecific interaction between the fusion partners.

### Coimmunoprecipitation and Western Blot Analysis

Co-IP assays were performed as described in a previous study (13). Briefly, transfected DEF cells were lysed in Pierce^®^ IP Lysis Buffer (Thermo Fisher) and incubated on ice for 1 h. Then, the lysate was transferred to a microcentrifuge tube and centrifuged at ~13,000 × g for 10 minutes to pellet the cell debris at 4°C. The supernatant was collected for protein concentration determination and further analysis. For each sample, 0.5 mL cell lysate was incubated with 10 μg of the indicated antibody and 1 mg of SureBeads Protein G (Thermo Fisher) at 4°C for 2 h. The SureBeads were washed 3 times with 1 ml PBST (PBS containing 0.1% Tween 20) and centrifuged at ~13,000 × g for 1 minute. Finally, the precipitates were fractionated by SDS-PAGE, and western blotting was performed with the appropriate antibody.

### Transient Replicon Activity Assay

Wild-type (WT) or mutant replicon (pACYC-duck-TMUV-NanoLuc-Rep) plasmids (400 ng each well) were transiently transfected into BHK-21 cells (seeded in 48-well plates). At 12, 24, 36, 48, 60, and 72 h p.t., cells were washed with PBS, lysed by adding 65 μL lysis buffer (Promega) and stored at −80°C until detection. The luciferase activities were measured using a Clarity luminescence microplate reader (BioTek) according to the manufacturer’s instructions.

### DNA Transcription and Transfection

Briefly, BHK-21 cells were seeded in 6-well plates containing 1 mL of DMEM and incubated overnight. Then, the BHK-21 cells were transfected with 4 μg plasmids for wild-type or mutant infectious clones and replicons. At the given time points, the cells were harvested for further study. The luciferase activity of the replicon was determined according to the manufacturer’s protocol.

### Viral Titers Detection

Viral titers were determined by the median tissue culture infectious dose 50 (TCID_50_) method in BHK-21 cells. Viral samples were serially diluted 10-fold in DMEM, and then 100 µL dilutions of the viral sample were distributed to each of 8 wells of a 96-well plate seeded with a monolayer of BHK-21 cells. After 120 h incubation at 37°C with 5% CO_2_, the presence of viruses was detected by assaying CPE using microscopy, and the viral titers were calculated according to the Reed-Muench method. For the growth curve assay, BHK-21 cells were infected with WT virus or with mutant virus at 100 TCID_50_. At the given time points, virus samples of the supernatants and cells were collected to determine the viral copies and viral titers.

### Plaque Assay

BHK-21 cells were seeded in 6-well plates containing 1 mL of DMEM and incubated overnight. Viral samples were serially diluted 10-fold in DMEM. Subsequently, the cells were infected with 200 µL of each dilution for 1 h at 37°C and 5% CO_2_ and swirled every 15 min to ensure viral attachment. After incubation, 2 mL of 0.75% methyl cellulose overlay containing 2% FBS and 1% penicillin/streptomycin was added to each well, and the plate was incubated at 37°C for 4 days. Then, the methyl cellulose overlay was removed, the plate was washed twice with PBS, fixed with 4% formaldehyde, and incubated at room temperature for 20 min. After removing the fixative, the plate was stained with 1% crystal violet for 1 min, we washed the cells carefully, and visible plaques were observed.

### Data Statistics

The statistical analyses were performed with GraphPad Prism 5 (GraphPad Software Inc., San Diego, CA, USA). The differences between the values were evaluated by Student’s t test. P < 0.05 was considered statistically significant, and all values are expressed as the mean ± SEM.

## Results

### Inhibition of Distinct Components From the RIG-I Pathway

**Figure 1:**
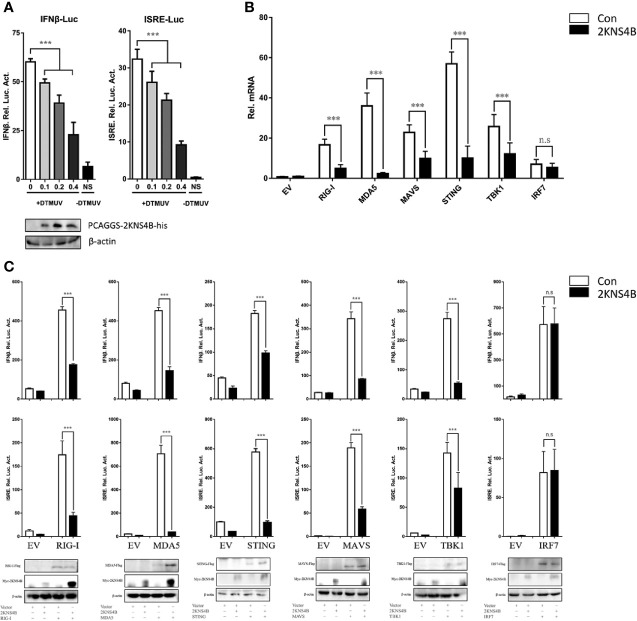
Duck TMUV 2KNS4B inhibits RIG-I-induced IFN signaling induced by the RIG-I pathway. **(A)** 2KNS4B inhibited duck TMUV-mediated IFN-β/ISRE promoter activity in a dose-dependent manner. Distinct doses of the 2KNS4B plasmid (100 ng, 200 ng or 400 ng/well) were transiently transfected into DEFs and subsequently cotransfected with the pRL-TK plasmid (40 ng/well), the pGL3-IFN-β-Luc plasmid (400 ng/well) or the pGL4-ISRE-Luc plasmid (400 ng/well). At 24 h posttransfection, the cells were infected with duck TMUV (25 µL containing 100 TCID_50_ per well), and the luciferase activities were measured at 36 h postinfection. **(B)** 2KNS4B inhibited the IFNβ mRNA induced by the RLR components. DEFs were transiently transfected with each of the pCAGGS plasmids expressing Flag-tagged components (400 ng/well) and His-tagged 2KNS4B (400 ng/well). After 24 h of transfection, the cells were harvested with 1 mL RNAiso Plus reagent for the detection of duIFNβ mRNA by RT-qPCR. All results were normalized to those of duβ-actin. **(C)** 2KNS4B inhibited the IFNβ/ISRE-Luc induced by the RLR components. The DEFs were transiently transfected with each of the above components (400 ng/well) and 2KNS4B (400 ng/well) and subsequently transfected with pRL-TK plasmid (40 ng/well), pGL3-IFNβ-Luc or pGL4-ISRE-Luc (400 ng/well). At 24 h posttransfection, the luciferase activities were measured. All data are represented as the mean ± SEM (n = 4). Significant differences were statistically analyzed by using the one-tailed unpaired t-test, indicated by ***(P < 0.001) and the non-significant indicated by n.s.

In a previous study, we determined the effects of each duck TMUV protein on the RIG-I signaling pathway. We found that the expression of NS2A, NS2B, and 2KNS4B proteins significantly inhibited the duck TMUV-triggered activation of IFN-β and the ISRE-Luc promoter, suggesting that 2KNS4B may act as an antagonist of IFN induction and alter the phases of the IFN response (13). We further verified the antagonistic effects of the 2KNS4B protein on the RIG-I signaling pathway in this study. We found that 2KNS4B significantly inhibited the virus-induced activation of the IFN-β and ISRE promoters in a dose-dependent manner (**Figure 1A**). Subsequently, we performed qPCR and reporter assays to screen candidate components of the RIG-I pathway that could potentially be targeted by 2KNS4B proteins for inhibition. As shown in **Figure 1B**, the expression of 2KNS4B inhibited the duIFN-β mRNA expression level induced by the components, except for IRF7. Moreover, plasmids expressing individual components from the RIG-I pathway (duRIG-I, duMDA5, duMAVS, duSTING, duTBK1, and duIRF7) were coexpressed with 2KNS4B and the luciferase reporter produced from a reporter plasmid harboring the IFN-β and ISRE promoters. Analysis of the luciferase activities showed that the expression of 2KNS4B significantly inhibited RIG-I-, MDA5-, MAVS-, STING- and TBK1-induced IFN-β and ISRE promoter activation (**Figure 1C**). Taken together, these results suggested that 2KNS4B suppressed IFN-β production by inhibiting TBK1 or its upstream step.

### Interaction of Duck TMUV 2KNS4B With STING

**Figure 2:**
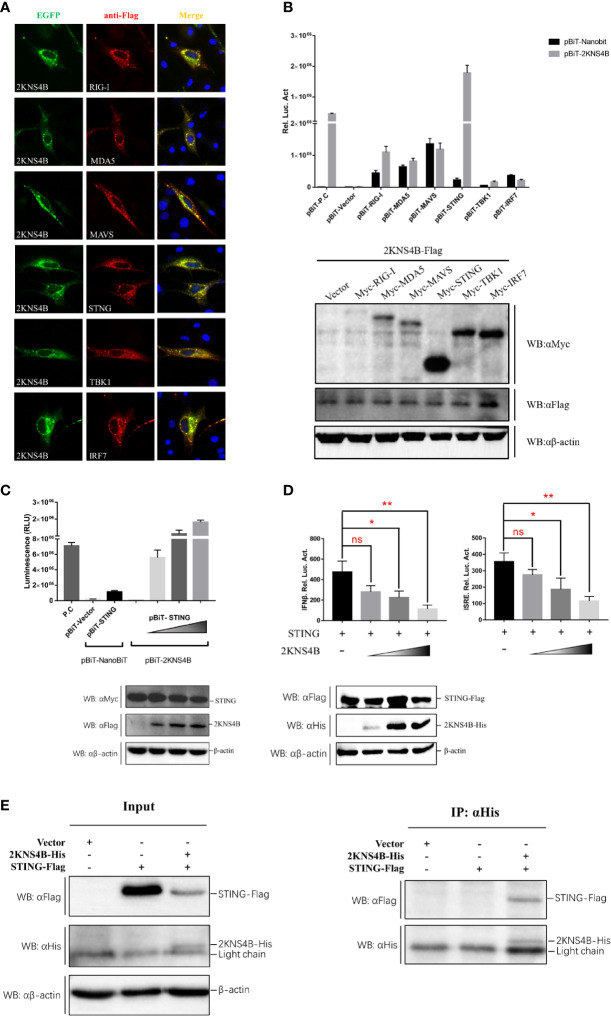
Duck TMUV 2KNS4B binds with STING. **(A)** IFA analysis. BHK-21 cells were cotransfected with each of the components (pCAGGS-RIG-I, MDA5, MAVS, STING, TBK1 and IRF7-Flag) (400 ng/well) and pCAGGS-2KNS4B-His (400 ng/well). At 24 h posttransfection, the cells were fixed in 4% paraformaldehyde overnight at 4°C for IFA assays. **(B)** NanoBiT PPI analysis. DEFs were cotransfected with pBiT-2KNS4B-SmBiT-Flag (400 ng/well) and each of the components (pBiT-RIG-I, MDA5, MAVS, STING, TBK1 and IRF7-LgBiT-Myc) (400 ng/well) for 20 h, then the luciferase activities were measured, and the protein expression levels were determined by western blotting. **(C)** Dose-dependent analysis of the 2KNS4B-STING interaction. DEFs were transiently transfected with the indicated amount of pBiT-2KNS4B-SmBiT-Flag plasmid (100 ng, 200 ng or 400 ng/well), along with 400 ng/well of pBiT-STING-LgBiT-Myc plasmid. The luciferase activities were measured at 20 h posttransfection, and protein expression levels were determined by western blotting. **(D)** Dose-dependent analysis of the inhibition of STING-mediated IFNβ-Luc/ISRE-Luc promoter activity by 2KNS4B. DEFs were cotransfected with the indicated amount of pCAGGS-2KNS4B-His plasmid (100 ng, 200 ng or 400 ng/well) and pCAGGS-STING-Flag (400 ng/well), along with pRL-TK plasmid (40 ng/well), pGL3-IFNβ-Luc (400 ng/well) or pGL4-ISRE-Luc (400 ng/well), and the luciferase activities were measured at 36 h postinfection. Protein expression levels were determined by western blotting. **(E)** Coimmunoprecipitation analysis of the 2KNS4B-STING interaction. DEF cells were cotransfected with pCAGGS-2KNS4B-His (800 ng/well) and pCAGGS-STING-Flag (800 ng/well). The cells were lysed in Pierce^®^ IP Lysis Buffer (Thermo Fisher) at 24 h posttransfection, and whole-cell extracts (WCEs) were loaded as input. The WCEs were incubated with 10 μg of the indicated antibody and 1 mg of SureBeads Protein (G) Finally, the precipitates were analyzed by SDS-PAGE and western blotting. All data are represented as the mean ± SEM (n = 4). Significant differences were statistically analyzed by using the one-tailed unpaired t-test, indicated by *(P < 0.05), **(P < 0.01) and the non-significant indicated by n.s.

To explore the molecular interactions of 2KNS4B in the IFN induction signaling pathway, IFA, NanoBiT-PPI assays and Co-IP assays were performed. We found that 2KNS4B might colocalized with duSTING or its upstream components (**Figure 2A**). Then, NanoBiT PPI assays showed that 2KNS4B could significantly interact with STING (**Figure 2B**), and 2KNS4B interacted with STING in a dose-dependent manner (**Figure 2C**). In addition, we observed that 2KNS4B significantly and sufficiently suppressed the duSTING-mediated activation of the IFNβ-Luc and ISRE-Luc promoters in a dose-dependent manner, but with increasing doses of NS2A, the expression level of STING was constant (**Figure 2D**). Moreover, the interaction of 2KNS4B with STING was further confirmed by a co-IP assay (**Figure 2E**). Collectively, these results suggested that 2KNS4B inhibited the duSTING-mediated activation of IFN-β/ISRE-Luc promotor activity by directly interacting with STING.

### Duck TMUV 2KNS4B Disrupts the STING-TBK1 Interaction

**Figure 3:**
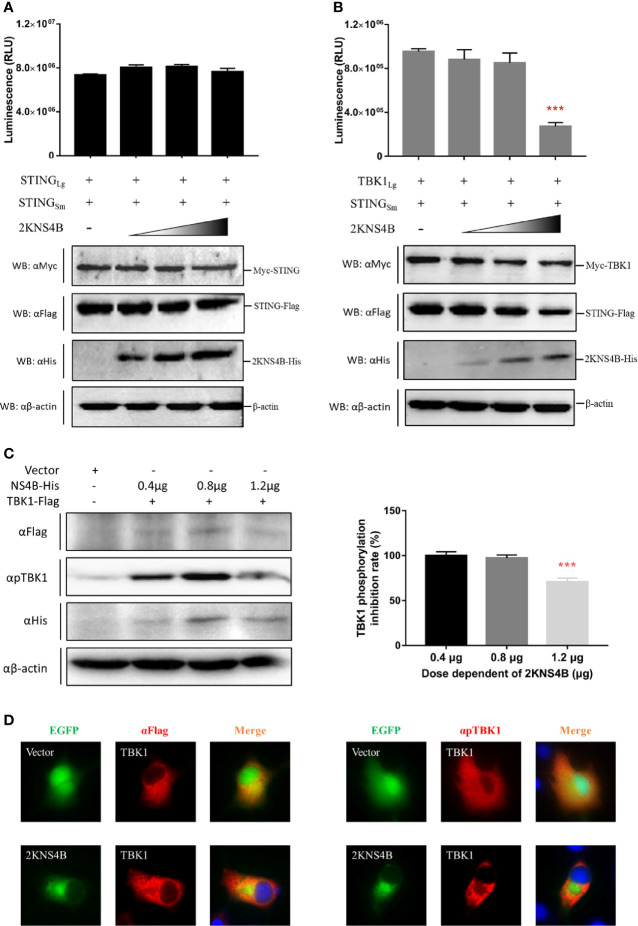
Duck TMUV 2KNS4B impairs the STING-TBK1 interaction, which leads to a reduction in TBK1 phosphorylation. **(A, B)** 2KNS4B competitively inhibits the interaction of STING with TBK1 but not with STING. pBiT-STING-SmBiT-Flag (400 ng/well) was cotransfected with pBiT-STING-LgBiT-Myc or pBiT-TBK1-LgBiT-Myc (400 ng/well) into DEF cells in the presence of pCAGGS-2KNS4B-His at different doses (100 ng, 200 ng or 400 ng/well). Luminescence was detected at 20 h posttransfection, and protein expression was measured by western blotting. **(C, D)** 2KNS4B suppresses the phosphorylation of TBK1. pCAGGS-TBK1-Flag (1200 ng/well) was cotransfected with pCAGGS-2KNS4B-His at different doses (400 ng, 800 ng or 1200 ng/well) into DEF cells for 24 h. The protein expression levels were determined by western blotting with the phospho-TBK1/NAK (Ser172) rabbit mAb (CST, America) **(C)**. pCAGGS-TBK1-Flag (1200 ng/well) was cotransfected with pEGFP-2KNS4B or pEGFP-Vector (1200 ng/well) into BHK-21 cells. At 24 h posttransfection, cells were fixed in 4% paraformaldehyde overnight at 4°C for IFA with the phospho-TBK1/NAK (Ser172) rabbit mAb (CST, America) **(D)**. All data are represented as the mean ± SEM (n = 4). Significant differences were statistically analyzed by using the one-tailed unpaired t-test, indicated by ***(P < 0.001).

To explore the molecular mechanism of 2KNS4B-mediated suppression of IFNβ induction, we examined whether 2KNS4B impairs the STING-STING and STING-TBK1 interactions. According to the results of the NanoBiT PPI assay, we found that 2KNS4B could significantly impair the formation of the STING-TBK1 complex in a dose-dependent manner, but not the STING-STING complex (**Figures 3A, B**). Furthermore, the phosphorylation of TBK1 was significantly reduced in the presence of a high dose of 2KNS4B, as shown by western blot and IFA (**Figures 3C, D**). These results indicated that the binding of 2KNS4B to STING inhibited the recruitment of TBK1 to STING, which might reduce TBK1 phosphorylation and inhibition of the IFN-β signaling pathway. Thus, 2KNS4B reduced the IFN-β signaling pathway by interacting with STING, which disrupted the formation of the STING-TBK1 complex.

### Domain Mapping of the Interaction Between Duck TMUV 2KNS4B and STNG

**Figure 4:**
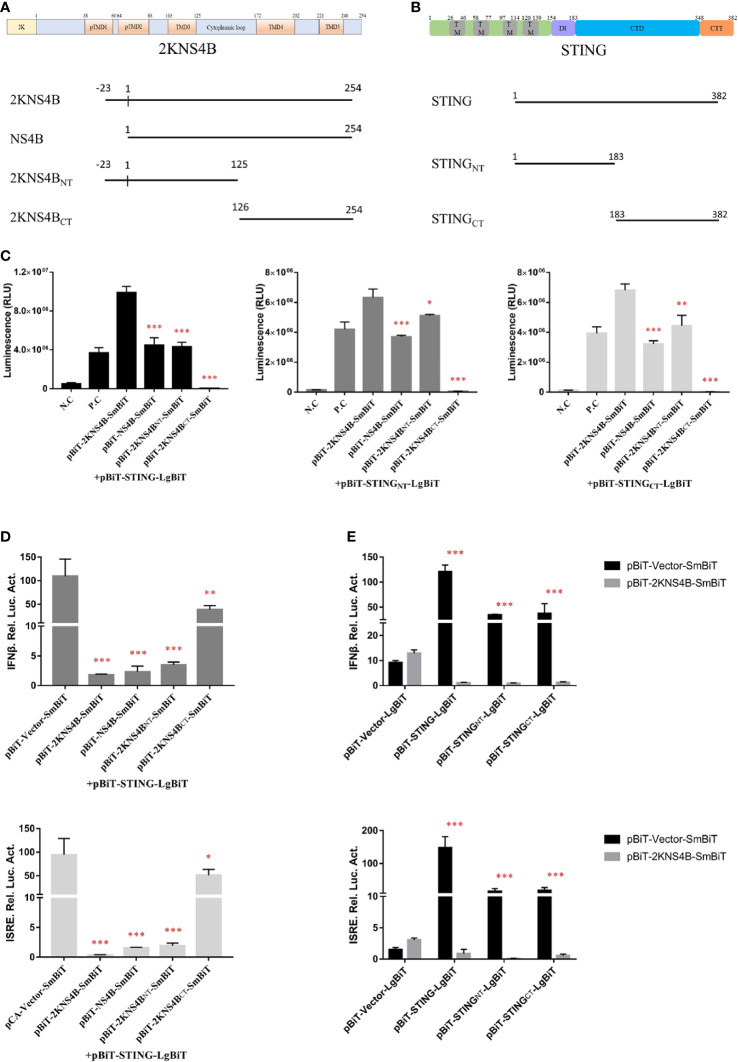
Domain mapping of the interaction between duck TMUV 2KNS4B and STING. **(A, B)** Schematic diagram of the 2KNS4B and STING deletions, the DI was represented as Dimerization interphase, CTD as C-terminal domain and CTT as C-terminal tail of STING. **(C)** DEFs were transiently transfected with 400 ng/well of each pBiT-LgBiT-Myc plasmid expressing STING and its truncations (STING_NT_ and STING_CT_), along with 400 ng/well of each pBiT-SmBiT-Flag plasmid with 2KNS4B and its truncations (NS4B, 2KNS4B_NT_ and 2KNS4B_CT_) or an empty vector. The luciferase activities were measured at 20 h posttransfection. **(D)** 2KNS4B and its truncations inhibited STING-induced IFNβ/ISRE-Luc activity. DEFs were cotransfected with pCAGGS-2KNS4B or 2KNS4B truncations-His (400 ng/well) and pCAGGS-STING (400 ng/well), along with pRL-TK plasmid (40 ng/well), pGL3-IFNβ-Luc (400 ng/well) or pGL4-ISRE-Luc (400 ng/well) for 24 h, and the luciferase activities were measured. **(E)** 2KNS4B inhibited STING and its truncation-induced IFNβ/ISRE-Luc activity. DEFs were cotransfected with pCAGGS-STING or STING truncations-Flag (400 ng/well) and pCAGGS-2KNS4B-His (400 ng/well) and subsequently transfected with pRL-TK plasmid (40 ng/well), pGL3-IFNβ-Luc (400 ng/well) or pGL4-ISRE-Luc (400 ng/well) for 24 h, and the luciferase activities were measured. All data are represented as the mean ± SEM (n = 4). Significant differences were statistically analyzed by using the one-tailed unpaired t-test, indicated by *(P < 0.05), **(P < 0.01) and ***(P < 0.001).

To investigate which domains of 2KNS4B and STNG are responsible for its interaction, three 2KNS4B truncations, NS4B (Δ2K), 2KNS4B_NT_ (1-125 aa) and 2KNS4B_CT_ (126-254 aa), were cloned into the pBiT-SmBiT vector with a Flag tag, while two STING truncations, STING_NT_ (1-183 aa) and STING_CT_ (183-382 aa), were cloned into the pBiT-LgBiT vector with a Myc tag (**Figures 4A, B**). DEFs were transiently transfected with 400 ng/well of each STING truncation plasmid, along with 400 ng/well of the three 2KNS4B truncation plasmids or an empty vector. The luciferase activities were measured at 20 h posttransfection (**Figure 4C**). We found that 2KNS4B_CT_ did not interact with STING or the STING truncations, while both the N-terminal and C-terminal regions of STING (STING_NT_ and STING_CT_) could interact with 2KNS4B. In addition, we also found that the 2K fragment is sufficient to affect the NS4B-STING interaction due to its function of proper cotranslational membrane insertion and protein folding. Then, a luciferase reporter assay was performed to determine the effect of 2KNS4B on STING-mediated IFN induction. As shown in **Figure 4D**, 2KNS4B and 2KNS4B_NT_ inhibited STING-mediated IFNβ/ISRE promotor activation, but NS4B_CT_ did not. In addition, 2KNS4B could inhibit STING- and STING truncation-mediated IFNβ/ISRE promoter activation (**Figure 4E**). Taken together, these results suggested that the N-terminus of 2KNS4B was essential for the interaction with STING and it inhibited the STING-triggered IFNβ signaling pathway.

### The 1-38 aa Region of Duck TMUV NS4B Is Critical for Its Interaction With STING and Its Inhibitory Effects

**Figure 5:**
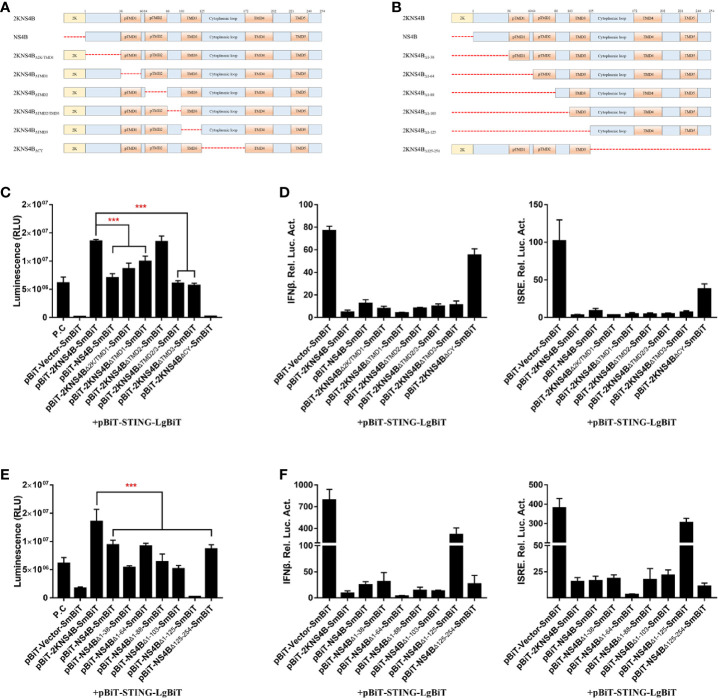
The 1-38 aa region of duck TMUV NS4B is essential for its interaction with STING and its inhibitory effect. **(A, B)** Schematic representation of full-length 2KNS4B and its deletions or truncations, and a 3xGS linker was inserted in the deletion region. **(C, D)** Deletion analysis of the interaction between 2KNS4B and STING and the inhibitory effect. DEFs were transiently transfected with 400 ng/well of each pBiT-LgBiT-Myc plasmid expressing STING, along with 400 ng/well of each pBiT-SmBiT-Flag plasmid with 2KNS4B (WT or deletions) or an empty vector. The luciferase activities were measured at 20 h posttransfection **(C)**. For the luciferase reporter assay, based on the above, the cells were subsequently transfected with the pRL-TK plasmid (40 ng/well), pGL3-IFNβ-Luc (400 ng/well) or pGL4-ISRE-Luc (400 ng/well). At 24 h posttransfection, the luciferase activities were measured **(D)**. **(E, F)** Truncation analysis of the interaction between 2KNS4B and STING and the inhibitory effect. DEFs were transiently transfected with 400 ng/well of each pBiT-LgBiT-Myc plasmid expressing STING, along with 400 ng/well of each pBiT-SmBiT-Flag plasmid with 2KNS4B (WT or truncations) or an empty vector. The luciferase activities were measured at 20 h posttransfection **(E)**. For the luciferase reporter assay, based on the above, cells were subsequently transfected with the pRL-TK plasmid (40 ng/well), pGL3-IFNβ-Luc (400 ng/well) or pGL4-ISRE-Luc (400 ng/well). At 24 h posttransfection, the luciferase activities were measured **(F)**. All data are represented as the mean ± SEM (n = 4). Significant differences were statistically analyzed by using the one-tailed unpaired t-test, indicated by ***(P < 0.001).

Moreover, to further confirm the interaction domain in the C-terminal region of NS4B, deletion (**Figure 5A**) and truncation (**Figure 5B**) analyses were performed. In the deletion assay, we found that the regions of 2KNS4B, besides TMD2, were sufficient to interact with STING and inhibit STING-mediated IFNβ/ISRE promoter activation. In addition, deletion of the cytoplasmic region of 2KNS4B aborted its interaction with STING and affected its inhibition of STING-mediated IFNβ/ISRE promoter activation (**Figures 5C, D**). In the truncation assay, we found that all truncations of 2KNS4B affected its interaction with STING and inhibited STING-mediated IFNβ/ISRE promoter activation (**Figures 5E, F**). These results revealed that changing the 2KNS4B residues may affect the spatial structure of 2KNS4B, which is responsible for the 2KNS4B-STING interaction, especially for the 1-38 aa region of NS4B.

### Alanine Scanning Mutagenesis in the 1-38 aa Region of the NS4B Protein

**Figure 6:**
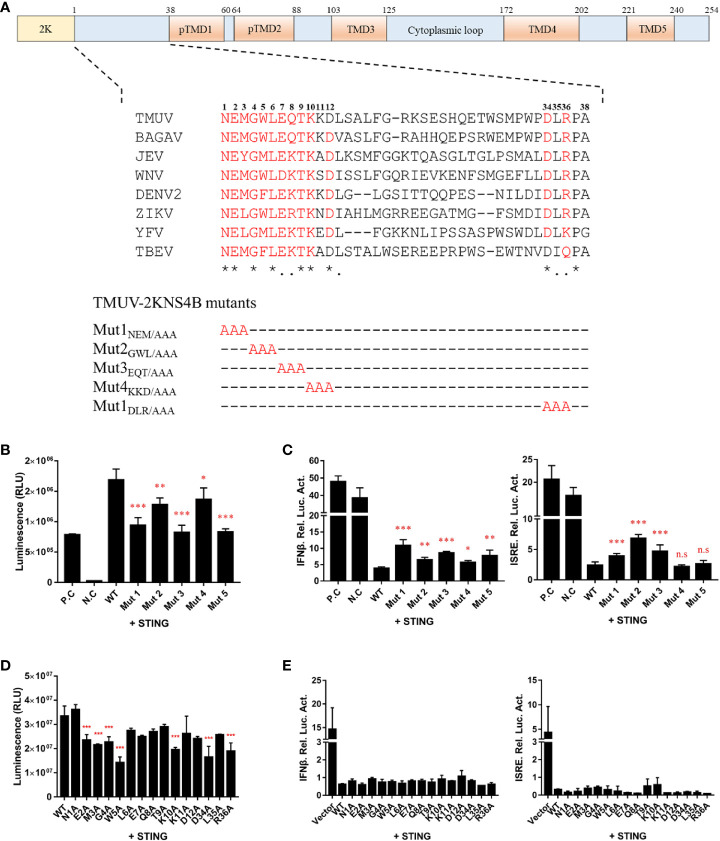
Alanine scanning mutagenesis of the 1-38 aa region of the duck TMUV NS4B protein. **(A)** Schematic diagram of alanine substitution mutations within the 1-38 aa region of NS4B. **(B, D)** Mutations of the 2KNS4B affect its interaction with STING. DEFs were transiently transfected with the pBiT-STING-LgBiT-Myc plasmid (400 ng/well) and pBiT-2KNS4B-SmBiT-Flag plasmid or its mutants or an empty vector (400 ng/well). The luciferase activities were measured at 20 h posttransfection. **(C, E)** Mutations of 2KNS4B affect the STING-induced IFNβ/ISRE-Luc activity. DEFs were cotransfected with 400 ng/well pCAGGS-His plasmid expressing 2KNS4B and its mutants and 400 ng/well pCAGGS-STING-Flag, along with pRL-TK plasmid (40 ng/well), pGL3-IFNβ-Luc (400 ng/well) or pGL4-ISRE-Luc (400 ng/well). At 24 h posttransfection, the luciferase activities were measured. Significant differences were statistically analyzed by using the one-tailed unpaired t-test, indicated by *(P < 0.05), **(P < 0.01), ***(P < 0.001) and the non-significant indicated by n.s.

In the flavivirus life cycle, the 2K fragment is cleaved off the N-terminus of NS4B by the host signal enzyme in the ER lumen, although the 2K fragment is critical for the NS4B-STING interaction. Thus, the 1-38 aa region of NS4B was chosen for further alanine substitution mutagenesis assays. To further determine which amino acids in the 1-38 aa region of NS4B contribute to its interaction with STING, multiple alanine substitution mutations in the 1-38 aa region of NS4B were introduced into pBiT-SmBiT with a Flag tag (**Figure 6A**). As shown in **Figure 6B**, the Mut 1, Mut 2, Mut 3 and Mut 5 mutants of NS4B markedly reduced the interaction with STING, and Mut 4 slightly inhibited the interaction with STING. In addition, a reporter assay showed that all NS4B mutations affected the inhibitory effects on STING-triggered IFNβ/ISRE promoter activation (**Figure 6C**). Subsequently, single alanine substitution mutations within the Mut 1 (N1A, E2A and M3A), Mut 2 (G4A, W5A and L6A), Mut 3 (E7A, Q8A and T9A), Mut 4 (K10A, K11A and D12A) and Mut 5 (D34A, L35A and R36A) mutants were generated in pBiT-SmBiT with a Flag tag. We found that residues E2, M3, G4, W5, K10 and D34 in NS4B were changed to “A” significantly reduced NS4B-STING interaction compared to WT, revealed that these residues are sufficient to interact with STING (**Figure 6D**). Moreover, these mutations inhibited STING-induced IFNβ/ISRE promoter activation to various degrees (**Figure 6E**). Taken together, these data indicate that residues E2, M3, G4, W5, K10 and D34 in NS4B are essential for its interaction with STING.

### Characterization of the Duck TMUV Genome RNA Replicon Disabling the NS4B-STING Interaction

**Figure 7:**
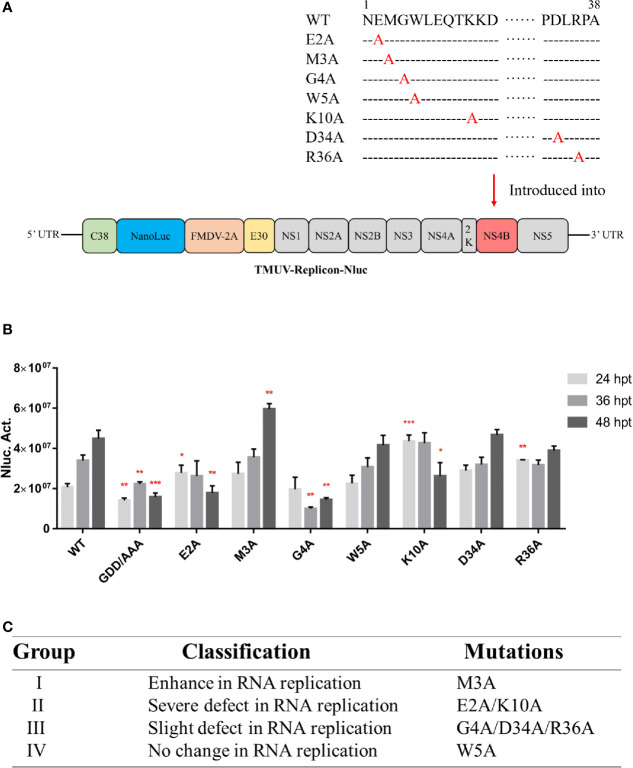
Characterization of the duck TMUV genome RNA replicon disabling the NS4B-STING interaction. **(A)** Schematic diagram of the duck TMUV NanoLuc luciferase reporter replicon. **(B)** The Nluc-duck TMUV reporter replicon (pAC-TVRep) was used to detect the replication efficiencies of the WT and NS4B-mutant replicons. Mutations in the NS4B-STING interaction sites were individually engineered into pAC-TVRep. Equal amounts of WT and mutant pAC-TVRep DNA were transfected into BHK-21 cells and assayed for luciferase activity at 24, 36 and 48 hpi. **(C)** Summary of the phenotypic effects of the NS4B-mutant replicon. Significant differences were statistically analyzed by using the one-tailed unpaired t-test, indicated by *(P < 0.05), **(P < 0.01) and ***(P < 0.001).

The nano luciferase reporter replicon of duck TMUV (pACYC-duck-TMUV-Replicon- NanoLuc, pAC-TVRepNluc) was used to further validate the mutational effect on viral RNA replication (**Figure 7A**). The luciferase activity kinetics of pAC-TVRepNluc-WT and pAC-TVRepNluc-NS5/GAA were detected. Mutations in the NS4B-STING interaction sites were individually engineered into pAC-TVRep. Equal amounts of WT and mutant pAC-TVRep DNA were transfected into BHK-21 cells and assayed for luciferase activity at 24, 36 and 48 hpi. As shown in **Figure 7B**, NS4B mutations could be classified into four groups based on the levels of the luciferase signals of the duck TMUV replicons. Group I mutants (M3A) enhanced viral replication. Group II mutants (E2A and K10A) had a severe defect in viral replication. Group III mutants (G4A, D34A and R36A) had slight defects in viral replication. Group IV mutants (W5A) exhibited the same luciferase profile as the WT (**Figure 7C**). Overall, our primary data suggest that residues involved in the NS4B-STING interaction also play important roles in viral RNA replication in some as yet unknown way.

### Characterization and Phenotypes of Duck TMUV Infectious cDNA Clones Disabling the NS4B-STING Interactions

**Figure 8:**
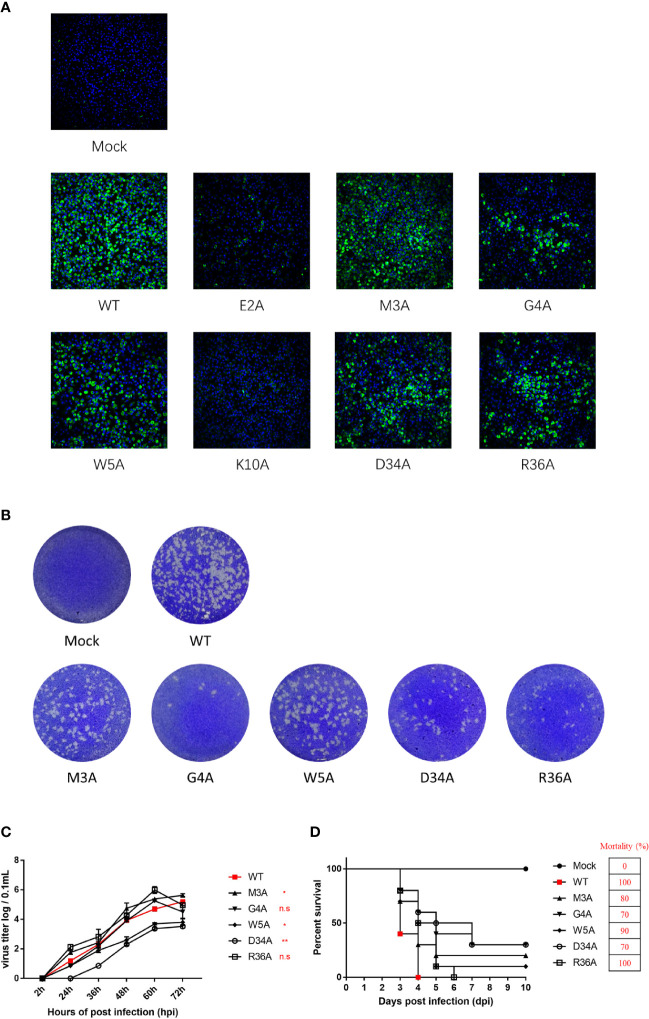
Characterization and phenotypes of duck TMUV infectious cDNA clones disabling NS4B-STING interactions. **(A)** Immunofluorescence analysis (IFA) of recombinant NS4B-mutant viruses. Equal amounts of WT and NS4B-mutant genomic RNAs were transiently transfected into BHK-21 cells. At 4 days posttransfection, the transfected cells were examined by IFA using the mouse anti-duck TMUV polyclonal antibody as the primary antibody. **(B)** Plaque morphology of WT and NS4B-mutant viruses. Viral samples were serially diluted 10-fold in DMEM. Subsequently, BHK-21 cells in 6-well plates were infected with 200 µL of each dilution for 1 h at 37°C and 5% CO_2_ and swirled every 15 min to ensure viral attachment. After incubation, 2 mL of 0.75% methyl cellulose overlay containing 2% FBS and 1% penicillin/streptomycin was added to each well, and the plate was incubated at 37°C for 4 days. Then, the methyl cellulose overlay was removed, and the plate was washed twice with PBS, fixed with 4% formaldehyde, and incubated at room temperature for 20 min. After removing the fixative, the plate was stained with 1% crystal violet for 1 min and washed carefully, and the visible plaques were observed. **(C)** Growth kinetics of WT and NS4B-mutant recombinant viruses in BHK-21 cells. BHK-21 cells were seeded in 12-well plates, and the cells were infected with 100 μL 100 TCID_50_ of WT or mutant virus. At the given time points, the cells were collected for the determination of viral titers. **(D)** Virulence of WT and NS4B-mutant recombinant viruses in duck embryos. Ten 9-day-old duck embryos per group were injected with 200 μL 100 TCID_50_ of rTMUV-WT and rTMUV-NS4B-mutant viruses by allantoic cavity inoculation. All viruses were diluted with PBS, and the mock group was injected with 200 μL of PBS. The eggs were incubated continually at 37°C, and the survival time of the infected embryos was recorded. Significant differences were statistically analyzed by using the one-tailed unpaired t-test, indicated by *(P < 0.05), **(P < 0.01) and the non-significant indicated by n.s.

To validate the results derived from the duck TMUV replicon, we engineered NS4B mutants into a full-length duck TMUV infectious cDNA clone (pACNR-rTMUV-WT). Equal amounts of WT and NS4B mutant genome length RNAs were transiently transfected into BHK-21 cells. At 4 days posttransfection, the transfected cells were examined by an IFA) using an anti-duck TMUV monoclonal antibody (**Figure 8A**). We found that the E2A and K10A mutants couldn’t be rescued, and the remaining mutants (M3A, G4A, W5A, D34A and R36A) yielded fewer IFA-positive cells than the WT. As expected, the morphology of the plaques exhibited the same results as the IFA (**Figure 8B**).

To compare the growth kinetics of the WT and NS4B mutant recombinant viruses in BHK-21 cells, they were incubated with P1 virus (we passaged the virus on BHK-21 cells for 1 consecutive round). At the indicated time points, the cells were harvested for detection of the virus titers. The results showed that the W5A and D34A mutant viruses exhibited lower titer levels than the WT. The G4A mutant virus replicated the same levels as WT at 60 hpi and before but showed lower titer levels than WT at 72 hpi. The highest peak titers of the M3A and R36A mutant viruses were higher than the WT (**Figure 8C**).

Moreover, the virulence of the WT and NS4B mutants were evaluated in duck embryos (**Figure 8D**). Ten 9-day-old duck embryos per group were injected with 200 μL 10^2^ TCID_50_ of P1 WT and NS4B mutant viruses. The duck embryos inoculated with the R36A mutant viruses exhibited similar mortality as the WT, whereas the M3A, G4A, W5A and D34A mutants were attenuated in duck embryos, especially for the G4A and D34A mutants that exhibited a lower mortality (70%) than WT. Taken together with the replicon results, these results suggested that the M3A, G4A, W5A and D34A mutants reduced viral virulence in duck embryos.

We also studied the genetic stability of the recombinant NS4B virus (attenuated in duck embryos) by passaging the virus in BHK-21 cells for 5 consecutive rounds (P0 to P5), and the complete genome sequence was obtained at P0 to P5. Our findings revealed that adaptive mutations emerged at P1 for G4A, P3 for WT and M3A, and P4 for W5A and D34A. Briefly, the G4A mutation in NS4B was reversed to A4G, and four other adaptive mutations (E-S150I, E-M349K, NS3-I435F and NS5-G643R) were obtained in the P5 G4A mutant. Furthermore, in addition to the original M3A, W5A and D34A, two adaptive mutations were obtained in the P5 M3A (E-M304K and NS5-G643R) and the P5 W5A (E-M304K, NS2B-N89N and NS5-G643R), and only one mutation was obtained in the P5 D34A (E-G392R). Interestingly, two adaptive mutations were detected in the WT, which probably generated some common changes (M304K in E and G643R in NS5) found in the rTMUV-WT virus, demonstrating that the M3A mutant displayed relative genetic stability at P0-P5 in BHK-21 cells.

## Discussion

Innate immune responses are the first line of the host’s defense against viral infection through the expression of hundreds of cytokines, especially type I IFNs. They are among the most important cytokines that play a critical role in the response to viral invasion. However, some flaviviruses encode multifunctional proteins that can be used to develop various strategies to antagonize the immune response of the host. Previous studies have reported that some flaviviruses can be recognized by TLRs and RLRs, which lead to the activation of type I IFN immune signaling pathways, whereas flavivirus nonstructural proteins can regulate the host innate immune response to improve viral replication efficiency (25–27). For example, WNV NS1 antagonizes interferon-β production by targeting RIG-I and MDA5 (28). The NS2B protein interacts with NS3 to form a stable complex that functions as a serine protease and is involved in the cleavage of polyproteins encoded by the viral genome (29). As previously reported, the DENV NS2B3 complex was shown to target and cleave MITA/STING, thereby inhibiting the IFNα/β-mediated innate immune response (30, 31). In the presence of DENV, NS2A and NS4B block RIG-I/MAVS signaling by inhibiting TBK1/IRF3 phosphorylation (10). Unlike DENV, YFV NS4B has also been shown to block RIG-I stimulation through an interaction with STING (32). Moreover, NS5 is known to establish a common mode of IFNα/β signaling antagonist for some flaviviruses by inhibiting the JAK/STAT pathway (33, 34).

In our recent study, we showed that duck TMUV NS2B3 cleaved and bound duck STING to subvert the induction of IFNβ (14). Additionally, we analyzed the ability of the 10 proteins encoded by duck TMUV to block the IFN system and found that the expression of NS2A, NS2B, and 2KNS4B resulted in robust IFN signaling inhibition. Further study found that NS2A competitively bound to STING with TBK1, suppressing IFN production and the subsequent phases of the IFN response (13). Therefore, different NS proteins of duck TMUV could antagonize IFN-β production through the common cellular components in RIG-I signaling pathway. However, the mechanism of how NS4B acts as an IFN antagonist in the inhibition of host immune responses remains to be further explored.

In this study, we verified that duck TMUV 2KNS4B could inhibit virus-induced IFNβ/ISRE promoter activities in a dose-dependent manner. Previous studies reported that flavivirus NS4B could inhibit RIG-I/MAVS signaling by blocking TBK1/IRF3 phosphorylation and inhibit IFN-mediated STAT1 phosphorylation to protect against the host immune response (10, 16, 17). Here, we found that duck TMUV 2KNS4B significantly inhibited RIG-I-, MDA5-, MAVS-, STING- and TBK1-induced IFNβ signaling but did not affect IRF7-induced IFNβ signaling. Subsequently, we identified that 2KNS4B specifically interacted with STING, resulting in a reduction in STING-mediated IFNβ/ISRE promoter activity. These results were consistent with duck TMUV NS2B3 and NS2A (13, 14) because the same cellular components could be targeted by different duck TMUV proteins to antagonize IFNβ production through the same or different strategies. We further found that 2KNS4B markedly suppressed the formation of the STING-TBK1 complex in a dose-dependent manner and significantly reduced subsequent TBK1 phosphorylation but did not affect the formation of the STING-STING complex. According to these results, we concluded that the interaction between 2KNS4B and STING inhibited the recruitment of TBK1 to STING, which subsequently reduced the phosphorylation of TBK1, leading to the inhibition of the IFNβ signaling pathway. Although HCV NS4B is different from flavivirus NS4B, consistent with our results, HCV NS4B could also target STING to prevent the interaction between STING and TBK1 upon stimulation, leading to blockade of interferon signaling (19). However, the molecular mechanism by which NS4B interferes with the formation of the STING-TBK1 complex requires further investigation. Additionally, there are several potential limitations of this study remaining to be further investigated. One is that the NS4B-STING interaction was only confirmed in the context of the ectopic expression systems due to the limitation of methods and materials. It’s necessary to confirm these interactions during the virus natural infection. The other is that we have performed all the experiments in primary duck embryo fibroblasts cells, which couldn’t be STING-KO cells. Thus, it’s necessary to test the ability of duck TMUV NS4B to prevent IFN induction through a STING mechanism in the STING-KO cells. Taken together, the molecular mechanism by which NS4B interacts with STING requires further investigation during the virus natural infection and in a STING-KO cells.

As the largest of the small hydrophobic NS proteins of flaviviruses, NS4B contains a similar membrane topology with five integral transmembrane segments (15, 35), which is responsible for the function of NS4B in viral replication and interactions with host proteins. For example, the N-terminus of NS4B is critical to suppress IFNβ signaling and is essential for interactions with host proteins (STING and/or PGK1). TMD3 and TMD5 of NS4B are able to inhibit the host RNA interference (RNAi) response. Both the cytoplasmic loop (amino acids 129-165) and the C-terminal region (amino acids 166-248) are essential for NS4B oligomerization (35). Additionally, the C-terminus of NS4B also functions as a binding site for KRT8. In our study, we identified that the N-terminal region of NS4B is essential for NS4B to interact with STING, including the N- and C-terminal regions of STING. Furthermore, we performed truncation and deletion assays to further identify the interaction region in the N-terminal region of NS4B between NS4B and STING; however, we found that the 2K fragment is essential for the NS4B-STING interaction and that changing the NS4B residues may affect the spatial structure of NS4B, which is critical to the interaction of NS4B-STING. Moreover, although the deletion of the cytoplasmic region of NS4B completely aborted its interaction with STING, we hypothesized that deletion the cytoplasmic region damages the spatial structure of NS4B, leading to the incorrect proper cotranslational membrane insertion and protein misfolding. We also found the difference in activity of Δ1-103 and Δ1-125 of NS4B, revealed that TMD3 is critical to NS4B-STING interaction. Nevertheless, according to the results of deletion and truncation assays, we focus on the 1-38aa region of NS4B, which markedly affected the NS4B-STING interaction. As shown in a previous study, the 2K fragment is cleaved off the N-terminus of NS4B by the host signal enzyme in the ER lumen, and this step is essential for proper cotranslational membrane insertion and protein folding (36, 37). Thus, we hypothesized that the 2K fragment affected the NS4B-STING interaction because deletion of the 2K fragment could disrupt the protein folding of NS4B in the ER lumen, making it difficult for NS4B to bind with STING. A previous study revealed that the 77-125 aa region of DEN-2 NS4B plays a critical role in the inhibition of IFN signaling (17), which is consistent with our results. The N-terminus of NS4B is essential for duck TMUV NS4B to inhibit IFN signaling. Although an increasing number of studies have provided evidence to prove that flavivirus NS4B targets STING, little is known about the interaction site between NS4B and STING and its function in virus replication. In our study, the 1-38 aa region of NS4B was subjected to multiple sequence alignment with several types of flaviviruses, and the conserved sites were analyzed by multiple and single alanine substitution assays. We found that E2, M3, G4, W5, K10 and D34 residues of NS4B were essential for the interaction with STING and its inhibitory effect on IFN signaling.

As a component of the viral replication complex with an essential role in viral replication and assembly, NS4B was recently reported to oligomerize to itself (18) as well as to interact with NS1 (38), NS2B (39), NS3 (40, 41) and NS4A (42, 43). Moreover, NS4B has also been reported to interact with host factors. Thus, NS4B was considered a potential antiviral target when treated with small-molecule inhibitors that block NS4B-virus protein or NS4B-host protein interactions, leading to the suppression of viral replication. The plant alkaloid lycorine was proven to inhibit the replication of WNV, YFV (44), and DENV (45), which was conferred by a V9M mutation in the viral 2K peptide. NITD-618 resistance is conferred by mutations P104 L and A119T in the TMD3 domain of NS4B, and abolishing the NS3-NS4B interaction could lead to the suppression of viral replication (46). Otherwise, flavivirus NS4B seems to be a mutational hotspot that is responsible for the development of live attenuated flavivirus vaccines. For example, a P38G substitution in WNV NS4B (isolate NY99) attenuated neuroinvasiveness in mice (47). In addition to participating in viral replication, NS4B is also expected to protect against the host immune response, which could be applied to the development of live attenuated vaccines and antiviral small-molecule inhibitors. To achieve this goal, we introduced NS4B mutants (E2, M3, G4, W5, K10, D34 and R36), which disabled the NS4B-STING interaction sites, into a duck TMUV replicon and a full-length duck TMUV infectious cDNA clone to validate the effect of these sites on viral replication. We found that the duck TMUV replicon with NS4B E2A and K10A mutations could not efficiently replicate in BHK-21 cells, resulting in a failure to rescue the virus. The G4A, D34A and R36A mutations decreased viral RNA synthesis and yielded fewer E protein IFA-positive cells than the WT. G4A mutant virus exhibited similar growth kinetics in BHK-21 cells as WT but showed significantly lower mortality (70%) than WT.

We found in our sequencing that two adaptive mutations had occurred at residue 150 (S150I) of the E protein and 643 (G643R, also occurred in the WT) in the P2 G4A mutant virus, resulting in replenishment of virus infectivity. The R36A mutant virus exhibited a higher peak titer than the WT and showed similar mortality (100%) as the WT, indicating that the R36A mutation was not sufficient to affect the infectivity of duck TMUV. For the D34A mutant virus, the virus titers were significantly decreased compared to WT and showed significantly lower mortality (70%), suggesting that the D34A mutation affects viral RNA synthesis and virus production, leading to attenuated virulence in duck embryos. In addition, the M3A and W5A mutations yielded fewer E protein IFA-positive cells and showed significantly lower mortality (80% and 90%, respectively) than WT. However, the M3A mutation enhanced viral RNA replication and showed significantly higher titer levels than WT at 48-72 hpi, whereas W5A exhibited a WT-like viral RNA synthesis phenotype and showed significantly lower titer levels than WT. It is possible that both the M3A and W5A mutations in NS4B may disrupt a critical step in virus assembly and release, and the virulence of both mutations was attenuated in duck embryos. Taken together, our data showed that the NS4B mutation M3A enhanced viral replication but attenuated the virulence of the virus in duck embryos, whereas the NS4B mutations W5A and D34A not only reduced viral production with effective replication but also attenuated the virulence of the virus in duck embryos. Thus, although M3A, W5A and D34A in duck TMUV NS4B could be considered new targets for the development of attenuated flavivirus vaccines and antiviral small-molecule inhibitors, further clinical testing is necessary to verify the safety and immunogenicity of these live attenuated vaccines.

In summary, we found that in the context of the overexpression systems, duck TMUV 2KNS4B significantly inhibited IFNβ and ISRE promoter activity by competitively binding to STING with TBK1, leading to a decrease in TBK1 phosphorylation. Remarkably, we further identified the amino acids at positions E2, M3, G4, W5, K10 and D34 to be essential for its interaction with STING and its inhibition of IFNβ induction using the Dual-Glo^®^ Luciferase Assay System and NanoBiT protein-protein interaction (PPI) assays. Subsequently, mutations at these positions were introduced into a duck TMUV replicon and an infectious cDNA clone. We found that the NS4B M3A mutant virus enhanced viral replication *in vitro* but attenuated the virulence of the virus *in vivo*, whereas the NS4B W5A and D34A mutants not only reduced viral production with effective replication *in vitro* but also attenuated the virulence of the virus *in vivo*. These findings indicate that the M3A, W5A and D34A mutations in NS4B may suppress virus assembly and/or release, resulting in attenuation of the virulence of the viruses. Thus, disabling NS4B-STING interaction sites could be considered a new approach to the rational attenuation of flaviviruses for the construction of vaccine candidates.

## Data Availability Statement

The original contributions presented in the study are included in the article/supplementary material. Further inquiries can be directed to the corresponding authors.

## Ethics Statement

The animal studies were approved by the Institutional Animal Care and Use Committee of Sichuan Agricultural University (No. SYXK(川)2019-189) and followed the National Institutes of Health guidelines for the performance of animal experiments.

## Author Contributions

SC and WZ designed the experiment. WZ, MZ, TL and BJ performed the experimental work. WZ and SC wrote the paper. WZ, JG, TH, MW, RJ, DZ, ML, XZ, QY, YW, SZ, XO, YL, LZ, YY, LP, and AC contributed to analysis the experimental data. All authors contributed to the article and approved the submitted version.

## Funding

This work was funded by grants from, the National Key Research and Development Program of China (2017YFD0500800), the Sichuan-International Joint Research for Science and Technology (2018HH0098), the China Agricultural Research System (CARS-42-17), and the Program Sichuan Veterinary Medicine and Drug Innovation Group of China Agricultural Research System (SCCXTD-2020-18).

## Conflict of Interest

The authors declare that the research was conducted in the absence of any commercial or financial relationships that could be construed as a potential conflict of interest.
